# Supplementary material for: Exophiala chapopotensis sp. nov., an extremotolerant black yeast from an oil-polluted soil in Mexico; phylophenetic approach to species hypothesis in the Herpotrichiellaceae family
Source: PLoS One. 2024 Feb 14;19(2):e0297232. doi: 10.1371/journal.pone.0297232 (PMC10866521; doi:10.1371/journal.pone.0297232)
Supplement: S1 Table — (PDF) [file pone.0297232.s001.pdf]

Supplementary Table 1. Partial tubulin beta chain mRNA deduced from the genome of *Exophiala chapopotensis* LBMH1013

```
>ECh_LBMH1013 Exophiala chapopotensis LBMH1013 tubulin beta chain
partial mRNA
tgcagtcttc gagagcgttt gcatggtcaa tactaagccc ctaaatggtt gttaacaaga
gcacaggcaa atcatctcag gcgaacantg gtcttgatgg ctctggagtg tatgtacatc
ctttccgtca gtgtggcggc cagactgaca gatttagcta ccatggcngc tacaagagtc
ttacccgaag atcacctngc tcattttgta caggcttctg gcaacaaata tgtccccgcg
ctgtgcttgt cgatctcgaa cccggtacca tggacgcncg ttcgagctgg acctttcggt
ctactnacaa tcgggagtaa agattacagg gttaacatgc tgtaggaaac aactgggcca
agggtcacta cactgagggc gctgangctg gtcgatcaag ttcttgatgt tgttcgacgt
gggctgagag ctgcgactgt ctgcaaggtt tccagattac ccactnctct tggaggtggt
accggtgccg gtatgggtac ttgttgatct ccaagatccg cgaagagttc ccagaccgca
tgatgngcca ctttctccgt cgttccatcc ccgaaggtct cgacaccgtc gtcgaaccat
acaacgccac gctctctgtt catcatcgta tgacccatag gtttggatac actcgtntga
ctgttttagg cactctacga tatctgtatg cgaccttgaa gctgtccaat ccttcatatg
gtgacctcaa ccacctnggt gtcagccgtc atgtccggcg ttaccacttg ctgcgtttcc
ccggtcagct gaactctgac ctgcgaaaat tggctgntca acatggttcc cttcccgcgt
ctccacttct tctggttggc ttcgcccctc tcaccagccg tggttcgtac tcgttcncgt
gcggtcacgg tgccagaact cactcaacaa atttcgaccc caagaacatg atggcggctt
cagatttccg aaatggntcg ctatttgact tgctcggcca tcttccgcgg caggtcagca
tgaaggaagt tgaagaccag atgcgcaatg tgcaganaca agaacagcag ctacttcgtc
gagtggattc ccacaacgtc cagaccgccc tgtgctctat tcctccgcgt ggcctcnaag
atgtcttcga ctttcgttgg caactctact tcatccaaga gctgttcaag cgtgtcggcg
atcaattcag tgccatngtt ccgtcgcaag gccttttttgc attggtacac tgggagggta
tggacgagat ggaattcacc gaggcagagt ccaacantga acgaccttgt ttccgaatac
cagcaatacc aaaagccagc atttccgagg gcg
```

```
DBLINK      BioProject: PRJNA821518
SOURCE      genome assembly Exophiala sp. LBMH1013
  ORGANISM  Exophiala sp. LBMH1013
            Eukaryota; Fungi; Dikarya; Ascomycota; Pezizomycotina;
            Eurotiomycetes; Chaetothyriomycetidae; Chaetothyriales;
            Herpotrichiellaceae; Exophiala.

REFERENCE
  AUTHORS   Ide-Pérez, M.R., Sánchez-Reyes,A., Folch-Mallol,J.L.,
and Sánchez-Carbente,M.R.
```

TITLE *Exophiala chapopotensis sp. nov., an extremotolerant black yeast from an oil-polluted soil in Mexico; phylophenetic approach to species hypothesis in the Herpotrichiellaceae family.*

Direct Submission

Centro de Investigacion en Biotecnologia, Universidad Autonoma del Estado de Morelos, Av. Universidad 1001, Cuernavaca, Morelos 62209,Mexico.
