# Supplementary material for: Exophiala chapopotensis sp. nov., an extremotolerant black yeast from an oil-polluted soil in Mexico; phylophenetic approach to species hypothesis in the Herpotrichiellaceae family
Source: PLoS One. 2024 Feb 14;19(2):e0297232. doi: 10.1371/journal.pone.0297232 (PMC10866521; doi:10.1371/journal.pone.0297232)
Supplement: S2 Table — (PDF) [file pone.0297232.s002.pdf]

Supplementary Table 2. Kmers frequency match between *Exophiala chapopotensis* LBMH1013and its closest phylogenetic neighbours

								Exophiala chapopotensis **LBMH1013 kmer frequency (%)**
**Kingdom**	**Phylum**	**Class**	**Order**	**Family**	**Genus**	**Species**	**Strain**	
Fungi	Ascomycota	Eurotiomycetes	Chaetothyriales	Herpotrichiellaceae	*Capronia*	*Capronia_coronata*	CBS_617_96	52.64
Fungi	Ascomycota	Eurotiomycetes	Chaetothyriales	Herpotrichiellaceae	*Exophiala*	*Exophiala_dermatitidis*	CBS_578.76	20.59
Fungi	Ascomycota	Eurotiomycetes	Chaetothyriales	Herpotrichiellaceae	*Exophiala*	*Exophiala_aquamarina*	CBS119918	11.70
Fungi	Ascomycota	Eurotiomycetes	Chaetothyriales	Herpotrichiellaceae	*Phialophora*	*Phialophora_macrospora*	BMU07676	4.97
Fungi	Ascomycota	Eurotiomycetes	Chaetothyriales	Herpotrichiellaceae	*Capronia*	*Capronia_epimyces*	CBS_606_96	4.38
