# Supplementary material for: Exophiala chapopotensis sp. nov., an extremotolerant black yeast from an oil-polluted soil in Mexico; phylophenetic approach to species hypothesis in the Herpotrichiellaceae family
Source: PLoS One. 2024 Feb 14;19(2):e0297232. doi: 10.1371/journal.pone.0297232 (PMC10866521; doi:10.1371/journal.pone.0297232)
Supplement: S1 Fig — Mash genomic distance, ANI, AAI, POCP. Analysis based on Correlations. Variances were computed as SS/N-1. Missing Data deletion: Casewise. No. of active Factors: 4; No. of active cases: 93. Eigenvalues: 2.84348 .830461 .314132. 011930. NSP: Non-Separable Data. (PDF) [file pone.0297232.s003.pdf]

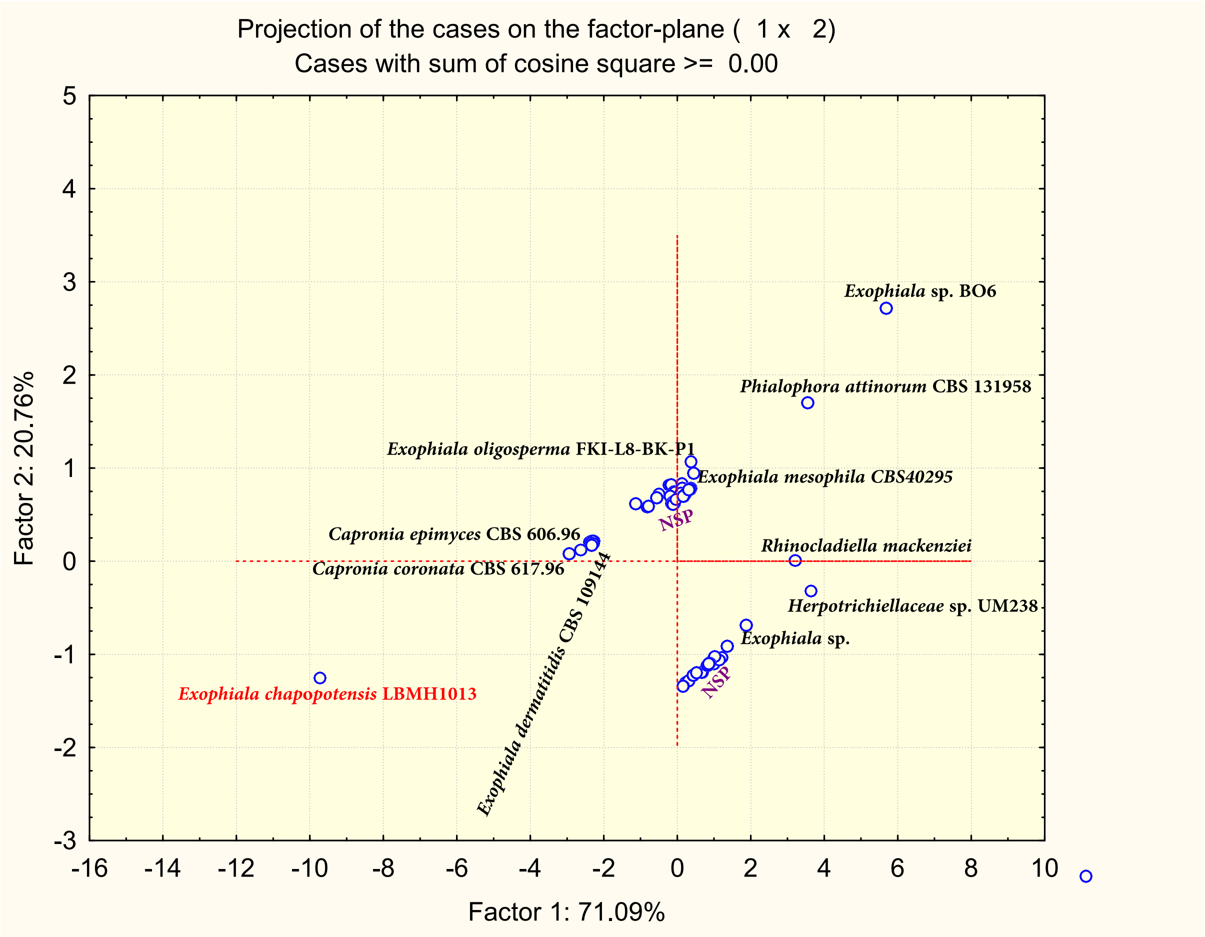

Supplementary Figure 1: Principal Components Analysis of the representatives of the *Herpotrichiellaceae* family taking the genome metrics as factors: Mash genomic distance, ANI, AAI, POCP. Analysis based on Correlations. Variances were computed as SS/N-1. Missing Data deletion: Casewise. No. of active Factors: 4; No. of active cases: 93.
Eigenvalues: 2.84348 .830461 .314132 . 011930
NSP: Non-Separable Data
