# Supplementary material for: Exophiala chapopotensis sp. nov., an extremotolerant black yeast from an oil-polluted soil in Mexico; phylophenetic approach to species hypothesis in the Herpotrichiellaceae family
Source: PLoS One. 2024 Feb 14;19(2):e0297232. doi: 10.1371/journal.pone.0297232 (PMC10866521; doi:10.1371/journal.pone.0297232)
Supplement: S2 Fig — (PDF) [file pone.0297232.s004.pdf]

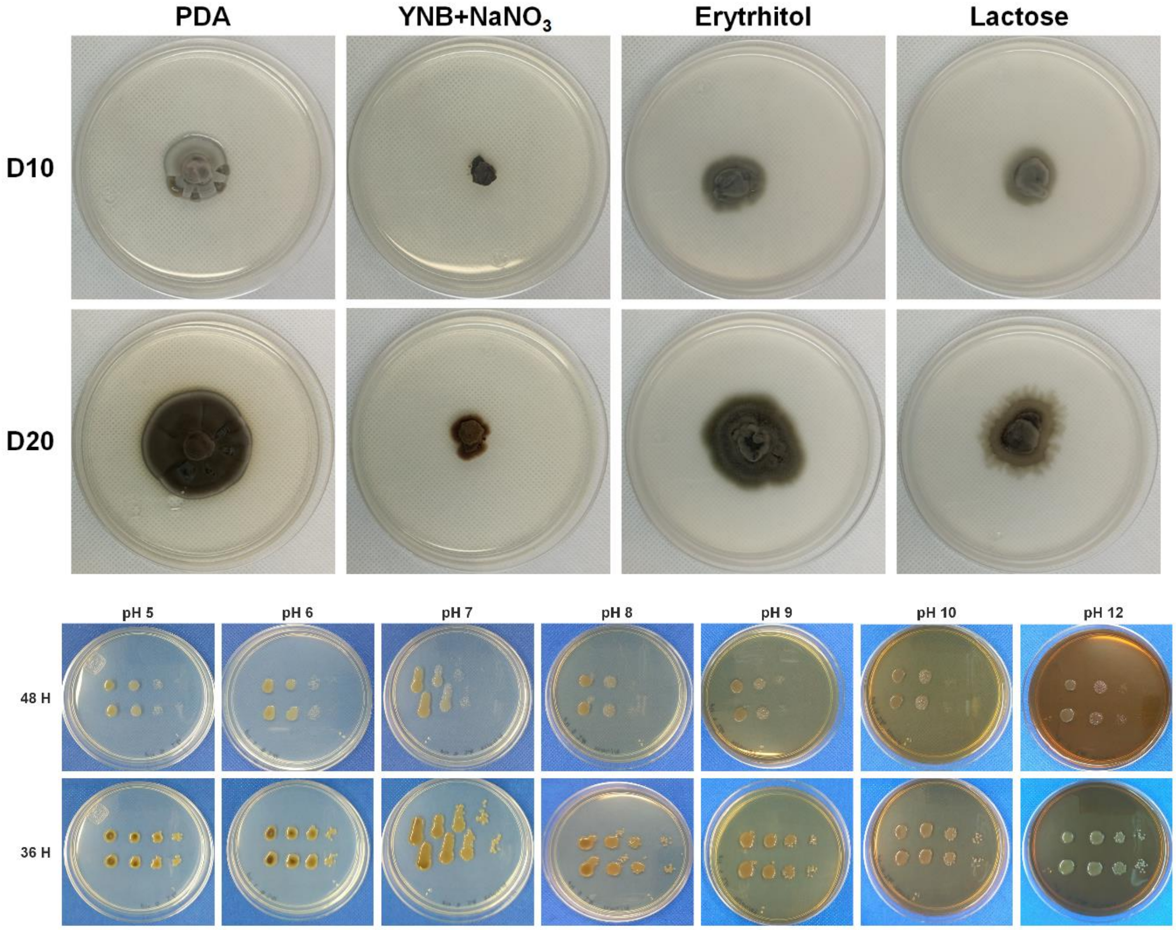

Supplementary Figure 2. Growth of strain LBMH1013 in different carbon sources (colonies at 10 and 20 days of growth are shown) and at different pH (48 and 36 hours).
