# Supplementary material for: Exophiala chapopotensis sp. nov., an extremotolerant black yeast from an oil-polluted soil in Mexico; phylophenetic approach to species hypothesis in the Herpotrichiellaceae family
Source: PLoS One. 2024 Feb 14;19(2):e0297232. doi: 10.1371/journal.pone.0297232 (PMC10866521; doi:10.1371/journal.pone.0297232)
Supplement: Attachment — Submitted filename: Review report of the research article Exophilla.docx [file pone.0297232.s005.docx]

Review report of the research article titled “ Exophiala chapopotensis sp. nov., an extremotolerant black yeast from an oil-polluted soil in Mexico; phylophenetic approach to species hypothesis in the Herpotrichiellaceae family”

The article is very important and useful for the scientific community. Being a Medical Scientist, we are reporting novel species time to time. There is a guideline and protocol we follow when we claim a new species. I have gone through the sequencing-based evidence what the authors documented, and I am convinced that the genetic evidence is enough to prove that *Exophiala chapopotensis* is a novel species and different from other Exophila species.

Any organism when we start looking it, we first grow them to understand their morphology, when we confirm the genus under microscope, we then compare the morphology with other species with same genus. We then go for different phenotypic experiments example pH, temp stress etc. to determine the variations from other related species and finally we do the electron microscopy to confirm that. In this work, the authors quoted that ref., 17 they claimed that described the phenotypic details which already been published. When I have gone through that article, I didn’t find enough phenotypic evidence to prove that this could be a novel species.

Hence, I suggest the authors, try to isolate another one or two of same organism in possible. Describe the detail of phenotypic analysis of novel species with closely related organisms and finally electron microscopy to prove that it is a novel species. When they will provide the requested documents, the article can be reconsidered for another revision. I am giving here two ref. article, and it will help the authors to understand hoe to report a novel fungal species.

A) Aime et al. IMA Fungus (2021) 12:11
B) Singh S et.al, *Frontiers in Cellular and Infection Microbiology*, July 2021 | Volume 11 | Article 686120
